# Sex differences in long-term effects of collagen-induced arthritis in middle-aged mice

**DOI:** 10.3389/fphys.2023.1195604

**Published:** 2023-06-28

**Authors:** Bernhard Maximilian Schuh, Kristína Macáková, Andrej Feješ, Tim Groß, Paulína Belvončíková, Jakub Janko, Dominik Juskanič, Samuel Hollý, Veronika Borbélyová, Emőke Šteňová, Michal Pastorek, Barbora Vlková, Peter Celec

**Affiliations:** ^1^ Faculty of Medicine, Institute of Molecular Biomedicine, Comenius University, Bratislava, Slovakia; ^2^ Jessenius-Diagnostic Center, Nitra, Slovakia; ^3^ Faculty of Medicine, Comenius University, Bratislava, Slovakia; ^4^ First Faculty of Medicine, Institute of Biophysics and Informatics, Charles University, Prague, Czechia; ^5^ 1st Department of Internal Medicine, Faculty of Medicine, University Hospital, Comenius University, Bratislava, Slovakia; ^6^ Faculty of Medicine, Institute of Pathophysiology, Comenius University, Bratislava, Slovakia

**Keywords:** autoimmune disease, autoantibodies, murine models, animal model limitations, synovial inflammation, disease severity, sex disparities, aging

## Abstract

**Introduction:** Rheumatoid arthritis (RA) is a chronic inflammatory disorder with high prevalence among middle-aged women. Collagen-induced arthritis (CIA) is the most widely used animal model of RA, however, sex differences and long-term effects of CIA in mice are poorly described in the literature.

**Aim:** Therefore, the present study aimed to analyze the long-term effects of CIA on the joints of middle-aged mice of both sexes and to describe potential sex differences.

**Materials and methods:** CIA was induced in middle-aged DBA/1J mice by immunization with bovine type II collagen and complete Freund’s adjuvant. Saline was administered to control mice. Arthritis score assessment, plethysmometry, and thermal imaging of the joints were performed weekly for 15 weeks. Locomotor activity, micro-computed tomography, joint histology and biochemical analyses were performed at the end of the experiment.

**Results:** Our results indicate a similar prevalence of arthritis in both sexes of mice—67% (8/12) of females and 89% (8/9) males with an earlier onset in males (day 14 vs. day 35). After the arthritis scores peaked on day 56 for males and day 63 for females, they steadily declined until the end of the experiment on day 105. A similar dynamics was observed in paw volume and temperature analyzing different aspects of joint inflammation. Long-term consequences including higher proteinuria (by 116%), loss of bone density (by 33.5%) and joint damage in terms of synovial hyperplasia as well as bone and cartilage erosions were more severe in CIA males compared to CIA females. There were no significant differences in locomotor activity between CIA mice and CTRL mice of any sex.

**Conclusion:** This is the first study to describe the long-term effects of the CIA model in terms of sex differences in DBA/1J mice. Our results indicate sex differences in the dynamics, but not in the extent of arthritis. An earlier onset of arthritis and more severe consequences on joints, bones and kidneys were found in males. The underlying immune pathomechanisms responsible for the limited duration of the arthritis symptoms and the opposite sex difference in comparison to RA patients require further investigation.

## 1 Introduction

Rheumatoid arthritis (RA) is a systemic chronic inflammatory autoimmune disease that primarily affects synovial joints (Smolen et al., 2018). The disease manifests with pain, swelling, stiffness, and progressive damage to the bone and cartilage of joints and is one of the most common chronic inflammatory diseases, with a prevalence of 0.5%–1% and a peak incidence at 50 years of age in developed countries (Silman and Pearson, 2002; Smolen et al., 2016; van der Woude and van der Helm-van Mil, 2018). The chance of developing RA is 2-3-fold higher in women than in men (Nilsson et al., 2021). Additionally, compared to men, women have on average higher disease activity and worse disability outcomes, whereas joint destruction is similar in both sexes (Sokka et al., 2009). The reason for this sexual dimorphism in RA is unknown and most likely multifactorial, however, sex hormones and their immunomodulatory effects may be among the key players (Cutolo et al., 2002; Kovacs and Olsen, 2011). Although the main characteristics of this disease are associated with joints, extra-articular involvement is common, and likely attributes to the higher morbidity and mortality seen in patients with RA compared to sex- and age-matched controls (Cojocaru et al., 2010; Løppenthin et al., 2019). The extra-articular manifestations of RA commonly encompass skin symptoms and involvement of the cardiovascular and respiratory system, but also renal involvement (Cojocaru et al., 2010; Chiu et al., 2015). The association between kidney disease and RA is thought to be linked to the disease either directly or indirectly, such as through the chronic inflammatory state or the use of nephrotoxic antirheumatic drugs (Karie et al., 2008; Kochi et al., 2016). Consequently, regular monitoring of renal function is of importance in RA patients, particularly for those at higher risk, such as the elderly and patients with comorbidities (Karie et al., 2008; Chiu et al., 2015). One of the markers that can be used for the detection of kidney damage in RA patients, is proteinuria, which is closely associated with glomerular or tubular damage (Niederstadt et al., 1999). Osteoporosis (OP) is another complication frequently observed in individuals with RA, particularly in women, with a prevalence of up to 50% in postmenopausal women diagnosed with RA (Llorente et al., 2020). Furthermore, articular bone erosions can be found in many patients with RA, and their presence indicates a more severe course of the disease. Although the exact mechanism behind articular bone erosions and OP in RA patients is unknown, enhanced osteoclast differentiation and osteoblast inhibition through receptor activator of nuclear factor κB ligand (RANKL) is a major precipitating factor (Schett & Gravallese, 2012; Llorente et al., 2020). However, the pathomechanism underlying RA remains elusive, and additional clinical and pre-clinical research is needed (Smolen et al., 2018). Therefore, animal models of RA provide a platform to explore the pathogenesis of the disease and assessment of the efficacy of potential anti-arthritic medications (Bendele et al., 1999). Although none of the animal models completely replicates the clinical pathology of human RA, they still provide valuable information about the development and progression of the disease (Asquith et al., 2009). Therefore, it is crucial to consider but also describe certain limitations between the animal models of RA and the actual human disease.

Collagen-induced arthritis (CIA) is the most widely used animal model of RA and resembles the disease by causing polyarthritis and erosion of cartilage and bone (Holmdahl et al., 2002; Asquith et al., 2009). Another similarity to RA is the linkage of susceptibility to the major histocompatibility complex (MHC) class two, leading to significant differences in the incidence of arthritis between certain mouse strains, with DBA/1J mice being one of the most susceptible (Adarichev et al., 2002). In 1977, Trentham et al. were the first to immunize rats with collagen and Freund’s adjuvant, resulting in the development of arthritis and an autoimmune response to cartilage (Trentham et al., 1977; Williams, 2004). Later, in 1980, Courtenay et al. translated the model into mice, and in recent years, it has been further improved by several other research teams (Courtenay et al., 1980; Williams, 2004). Currently, CIA is induced by immunization of mice through intradermal injection with an emulsion containing collagen type 2 (CII), usually of bovine, porcine, or chicken origin, and complete Freund’s adjuvant (CFA), leading to the production of large numbers of autoreactive antibodies targeting the articular cartilage (Durie et al., 1994; Brand et al., 2007). This immune response leads to swelling of mouse paws after approximately 3 weeks that decreases again around the eighth week post-immunization (Brand et al., 2007). Therefore, the CIA model is usually observed for 5–8 weeks, ending with the peak of arthritis severity and incidence. Most often, young male mice (8–12 weeks old) are used because of the potential influence of female sex hormones and age-related systemic changes on the model (Brand et al., 2007). However, there are also controversies about low to no incidence of arthritis in female mice, lower susceptibility of aged mice, and the development of spontaneous arthritis in male DBA/1J mice older than 4 months (Holmdahl et al., 1985; 1986; Brand et al., 2007; Braem et al., 2012; Marinov et al., 2022). However, the course of CIA and its long-term effects on the joints of middle-aged mice need to be investigated in both sexes to better describe the similarities and limitations to the clinical relevance of the CIA model for human RA.

Therefore, the aim of this study was to analyze the course and the long-term effects of CIA in middle-aged male and female mice to mimic the most frequent onset of RA in humans. We hypothesized that the female sex will worsen the progress of the CIA model and increase the risk of long-term consequences.

## 2 Materials and methods

### 2.1 Animals and initiation of collagen-induced arthritis

In this study, 10 months old female and male DBA/1J mice were used (Jackson Laboratory, JAX stock #000670, Bar Harbor, Maine, United States). The individual groups were composed of 12 female CIA mice and five female control mice, as well as nine male CIA mice and five male controls, respectively. Animals were group-housed (three to four per cage) in polycarbonate cages (36.5 cm × 20.5 cm × 14 cm) and kept under standard laboratory conditions (temperature 22°C ± 2°C, humidity 55 ± 10%, and 12:12 light-dark cycle). Animals had *ad libitum* access to food (standard diet for mice KMK-20, Eypy, Czech Republic) and water.

CIA was induced according to the standard protocol through an intradermal injection of an 100 μL of 1:1 emulsion containing bovine CII (Chondrex, Redmond–Woodinville, United States) and CFA (Chondrex, Redmond–Woodinville, United States) with a *M. tuberculosis* concentration of 2 mg/ml, into the base of the tail (Brand et al., 2007). The control group consisted of aged matched mice of both sexes and received a sham injection (0.9% NaCl solution) administered subcutaneously into the base of the tail.

All experimental procedures were approved by the Ethics Committee of the Institute of Molecular Biomedicine, Comenius University, Bratislava, and have been conducted in accordance with the EU Directive 2010/63/EU and Slovak legislation.

### 2.2 Visual assessment of the severity of arthritis and measurement of paw volume

The severity of arthritis was evaluated visually using a scoring system ranging from 0 to 4 points for each paw separately. For scoring, three types of joints were observed: Interphalangeal, metacarpophalangeal, and carpal for the front or tarsal for the back paws. Score 0—normal/physiologic state, Score 1—One of the three above-mentioned joint types has redness and swelling. Score 2—Two joints from the above-mentioned joint types have redness and swelling. Score 3—All three joint types have redness and swelling. Score 4—Achievement of maximal redness and swelling of the entire paw. The arthritis score of each mouse was obtained by the sum of the scores obtained from each paw (Brand et al., 2007).

For the evaluation of the paw swelling, the paw volumes were measured on the principle of volume displacement using a plethysmometer (Ugo Basile, Comerio VA, Italy), specifically by dipping the mouse paw up to the carpal joint in the front paw and the tarsal joint in the back paw. The technical variability for the plethysmometer measurements was <5%.

### 2.3 Assessment of body temperature and temperature of paws

Infrared thermography (IRT) was deployed as an additional method of assessing the severity of paw inflammation. Therefore, a thermal camera was used to assess the body temperature and temperature of all four paws (Teledyne FLIR-E64501, Wilsonville, OR, United States). The measurements were conducted in a room with the temperature consistently maintained at 25°C, and the mice were habituated for 30 min prior to the measurements being performed. Following the habituation period, the animals were placed into an induction chamber with a continuous flow of isoflurane (3%) mixed with oxygen (97%). Once adequately anesthetized, the animals were transferred to a tubing mask system (3% isoflurane mixed with oxygen) to obtain thermographic images. To reduce potential extrinsic bias, mice were only handled by touching the tip of their tail. The distance between the camera and the mouse was 20 cm. The thermal images were analyzed using the FLIR Tools software. To determine the temperature of the paws, elliptical regions of interest (ROI) were placed over the paws of the mice. Body temperature was evaluated using an elliptical ROI encircling the entire body of mice, excluding the paws. For normalization, the temperature of each paw was divided by the mean body temperature of the corresponding picture. The temperature index was adopted and modified from another team, which had established the use of thermography in the CIA model, as follows (Nosrati et al., 2020):

$$\text{Temperature}\;\text{index}:\bar{x}\;\left(\frac{TFL}{Tback}+\frac{TFR}{Tback}+\frac{THL}{Tback}+\frac{THR}{Tback}\right)$$

Tback: mean body temperature of the back of the mouse; TFL: temperature of the front left paw; TFR: temperature of the front right paw; THL: temperature of the hind left paw; THR: temperature of the hind right paw.

### 2.4 Radiological analysis/micro-computed tomography (CT)

To assess the bone density, juxta-articular bone density, which is typically affected in RA, was measured. For scanning of the mouse paw, Micro-CT (IVIS Spectrum CT, Caliper Life Sciences, United States) x-ray lamp with copper filter operated on 50 kV voltage and 1 mA s current, with an isotropic voxel size 0.15 mm × 0.15 mm × 0.15 mm, was used. Two readers experienced in musculoskeletal anatomy performed measurements of all available micro-CT scans using a standard DICOM viewer (TomoCon Viewer, Tatramed, Slovakia) and medical-grade diagnostic monitors. Readers were not involved in the scanning process and the DICOM header did not include information about inclusion in the intervention or control arm. The measurement consisted of placing an elliptical ROI (perimeter range 0.9–1 mm) inside the trabecular bone of the second metatarsal head of the right hind paw, as close to the joint surface as possible, while avoiding the cortical bone. Readers performed manual windowing and zooming to differentiate between trabecular and cortical bone parts.

### 2.5 Open field test

At the end of the experiment, the open field test was conducted to assess locomotor activity of mice. Open field test was performed in PhenoTyper cages with a square arena (45 cm × 45 cm) virtually divided into central and border zones. Mice were habituated for 30 min to the testing room environment. Following habituation, mice were individually placed in the center of the arena for 10 min. For evaluation of locomotor activity in mice, the parameter of total distance moved (cm) was observed. The behavior of mice was recorded and analyzed using EthoVision XT 16.0 (Noldus Information Technology, Wageningen, Netherlands).

### 2.6 Proteinuria

At the end of the experiment, the urine of the mice was collected using metabolic cages for 4 hours and stored at −20°C until further analyses. Proteinuria was analyzed using the Pyrogallol Red method (Watanabe et al., 1986). Briefly, 10 µL of urine samples and standards (bovine serum albumin) were mixed with 300 µL of pyrogallol red solution. After incubation at 37°C for 15 min, absorbance was measured at 595 nm.

### 2.7 Plasma concentrations of sex hormones and inflammatory cytokines

To assess plasma testosterone and estradiol concentrations, blood samples were collected from the retro-orbital plexus at the end of the experiment. Blood samples were centrifuged at 2000 g for 5 min. The concentration of testosterone and estradiol in plasma was measured using the commercially available ELISA kit (DRG Diagnostic, Marburg, Germany). The intra-assay and inter-assay coefficients of variation were below 5% and 10%, respectively. The assessment of circulating concentrations of inflammatory cytokines (TNFα, IL-1α, IL-10, IL-6, IL-1β, IL-23, INF-ℽ, INF-β, GM-CSF, IL-17A, IL-27, and MCP-1) was conducted using the LEGENDplex™ Mouse Inflammation Panel (Biolegend, Cat#740150, San Diego, CA, United States) in V-bottom plates and performed according to the instructions of the manufacturer using flow cytometer (DxFLEX, Beckman Coulter Life Sciences, United States). Plasma cytokine concentrations were calculated from the calibration curves of the standard solutions.

### 2.8 Histological analysis

The paws of mice were initially fixed in 4% formaldehyde, followed by decalcification for 1 month (14% EDTA, pH = 7.2 at 4°C) and embedded into paraffin blocks. All blocks were cut on a Hyrax M40 rotary microtome (Zeiss, Germany) and tissue sections were placed on glass slides (Waldemar Knittel, Germany) and stained with standard hematoxylin-eosin staining (H&E) to examine inflammation and joint damage. The samples were described and evaluated in a light-microscopic picture using an optical microscope (Olympus Provis BX40, Japan). For the evaluation of pathological changes in joints an established scoring system was deployed, which considered the following parameters: infiltration of inflammatory cells, synovial hyperplasia, as well as erosions to cartilage and bone. A more detailed and comprehensive description of the used scoring system can be found in Supplementary Table S1 (Li et al., 2021).

### 2.9 Statistical analysis

Statistical analysis was performed using GraphPad Prism version 9 (GraphPad Software, Inc., CA, United States). Two-way analysis of variance (2-way ANOVA, independent factors: time and group) or one-way ANOVA and the Bonferroni-corrected *post hoc t*-test were used. The cumulative incidence of arthritis score was estimated using the Kaplan-Meier method and log-rank test as the presence of any first arthritis signs. The area under the curve (AUC) was calculated from the dynamics of arthritis score, front and back paws swelling, and temperature index. For evaluation of plasma sex hormones, Student’s t-test (two-sided) was used. Evaluation of plasma cytokines and histological results were analyzed using 2-way ANOVA (independent factors: sex and treatment). The relationship between arthritis score and swelling of the paws was analyzed using Pearson’s correlation coefficient. *p*-values lower than 0.05 were considered statistically significant. Data are presented as mean plus standard deviation (SD) or standard error of the mean (SEM).

## 3 Results

### 3.1 Arthritis score and dynamics of CIA

**FIGURE 1:**
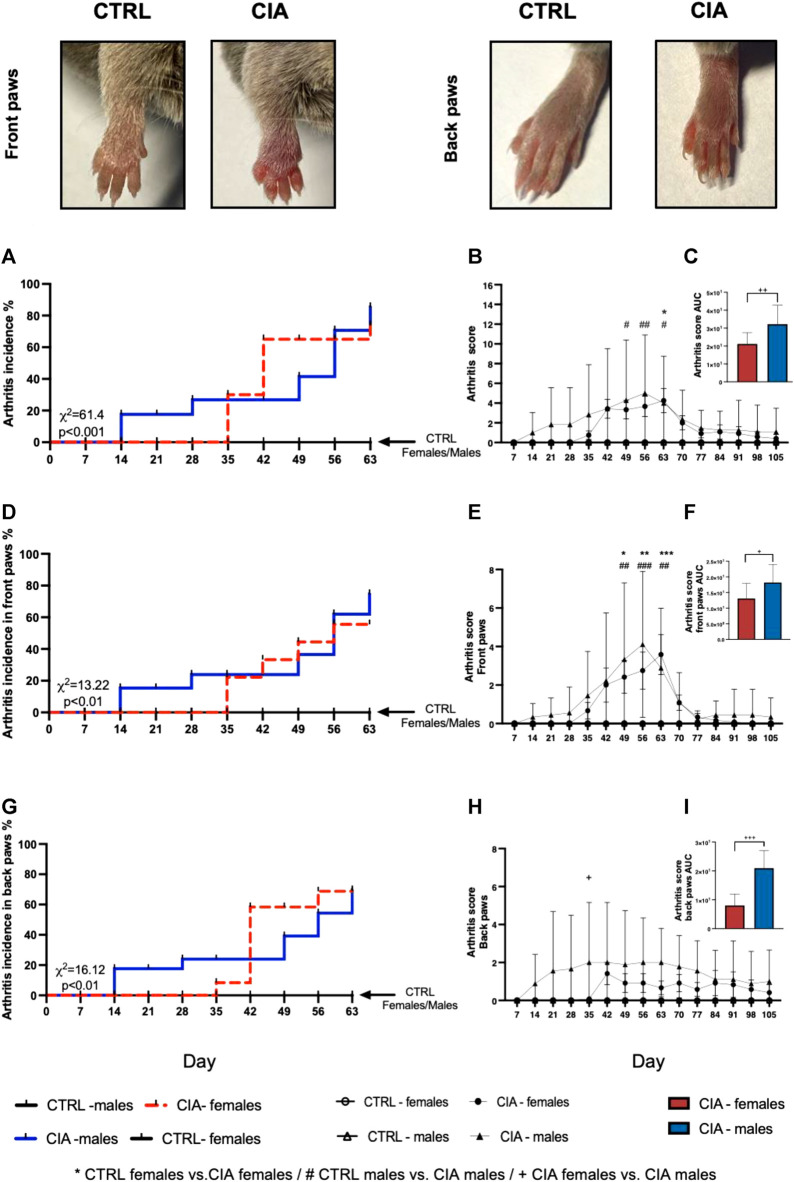
Incidence of arthritis signs followed by the dynamics of arthritis score. Representative pictures of the development of the arthritis signs, including redness, swelling and thickness of CIA mice paws compared to CTRL mice paws (taken on day 56, representing a score 4 for the front paw and a score 2 for the back paw). **(A)**—Incidence of arthritis signs for front and back paws according to the sex of mice. **(B)**—Dynamics of arthritis scores for the front and back paws. **(C)**—Dynamics of arthritis scores for front and back paws in the area under the curve between male and female CIA mice. **(D)**—Incidence of arthritis for front paws according to the sex of mice. **(E)** - Dynamics of arthritis scores for the front paws. **(F)**—Dynamics of arthritis scores for front paws in the area under the curve between male and female CIA mice. **(G)** Incidence of arthritis signs for back paws according to the sex of mice. **(H)** Dynamics of arthritis scores for the back paws. **(I)**—Dynamics of arthritis scores for back paws in the area under the curve between male and female CIA mice. [CTRL-females (*n* = 5), CIA-females (*n* = 12), CTRL-males (*n* = 5), CIA-males (*n* = 9)]. (*p* < 0.05 = *, *p* < 0.01 = **, *p* < 0.001 = ***, *p* < 0.05 = #, *p* < 0.01 = ##, *p* < 0.001 = ###, *p* < 0.05 = +, *p* < 0.01 = ++, *p* < 0.001 = +++).

Following collagen injection, first CIA symptoms developed 21 days earlier in males than in females (day 14 vs. day 35) (χ^2^ = 61.4; *p* < 0.001; Figure 1A). Overall, 67% (8/12) of the female and 89% (8/9) male mice developed signs of arthritis during the course of the experiment. The 2-way ANOVA showed a significant effect of time factor (F = 3.28; *p* < 0.001), but not group factor (F = 2.66; *p* = 0.06) on the dynamics of the arthritis score (Figure 1B). The CIA females displayed significantly higher arthritis scores on day 63 compared to CTRL females, while the CIA males displayed significantly higher arthritis scores on days 49, 56, and 63 compared to CTRL males (all: *p* < 0.05; Figure 1B). Also, the AUC from the dynamics of the arthritis score was significantly higher in CIA males compared to CIA females, indicating sex differences in the development of arthritis (t = 2.995; *p* < 0.01, Figure 1C). The first arthritis signs in front paws were developed 14 days post-immunization in CIA males, while in CIA females on day 35 post-immunization (*p* < 0.001; Figure 1D). There was a significant effect of time (F = 5.03; *p* < 0.001) and group (F = 3.54; *p* < 0.05) factors on the front paw arthritis score during observation. Both, CIA female and male mice displayed significantly higher arthritis scores on their front paws on days 49, 56, and 63 compared to their control groups (all: *p* < 0.05; Figure 1E). The AUC from the dynamics of the arthritis score in front paws was significantly higher in CIA males compared to CIA females (t = 2.186; *p* < 0.05; Figure 1F). CIA females developed first arthritis signs on front paws on day 35 of the experiment, while CIA males 14 days post-immunization (χ^2^ = 13.22; *p* < 0.01; Figure 1D). The first arthritis signs on back paws developed in the CIA females 35 days, while in CIA males 14 days post-immunization (𝜒2 = 16.12; *p* < 0.01; Figure 1G). The arthritis score of back paws was affected by group factor (F = 3.71; *p* < 0.05), and back paw score was higher in CIA males compared to CIA females on day 35 after collagen injection (*p* < 0.05; Figure 1H). The AUC calculated from dynamics of the arthritis scores of back paws was significantly higher in CIA males compared to CIA females (t = 5.949; *p* < 0.001; Figure 1I).

### 3.2 Temperature index

**FIGURE 2:**
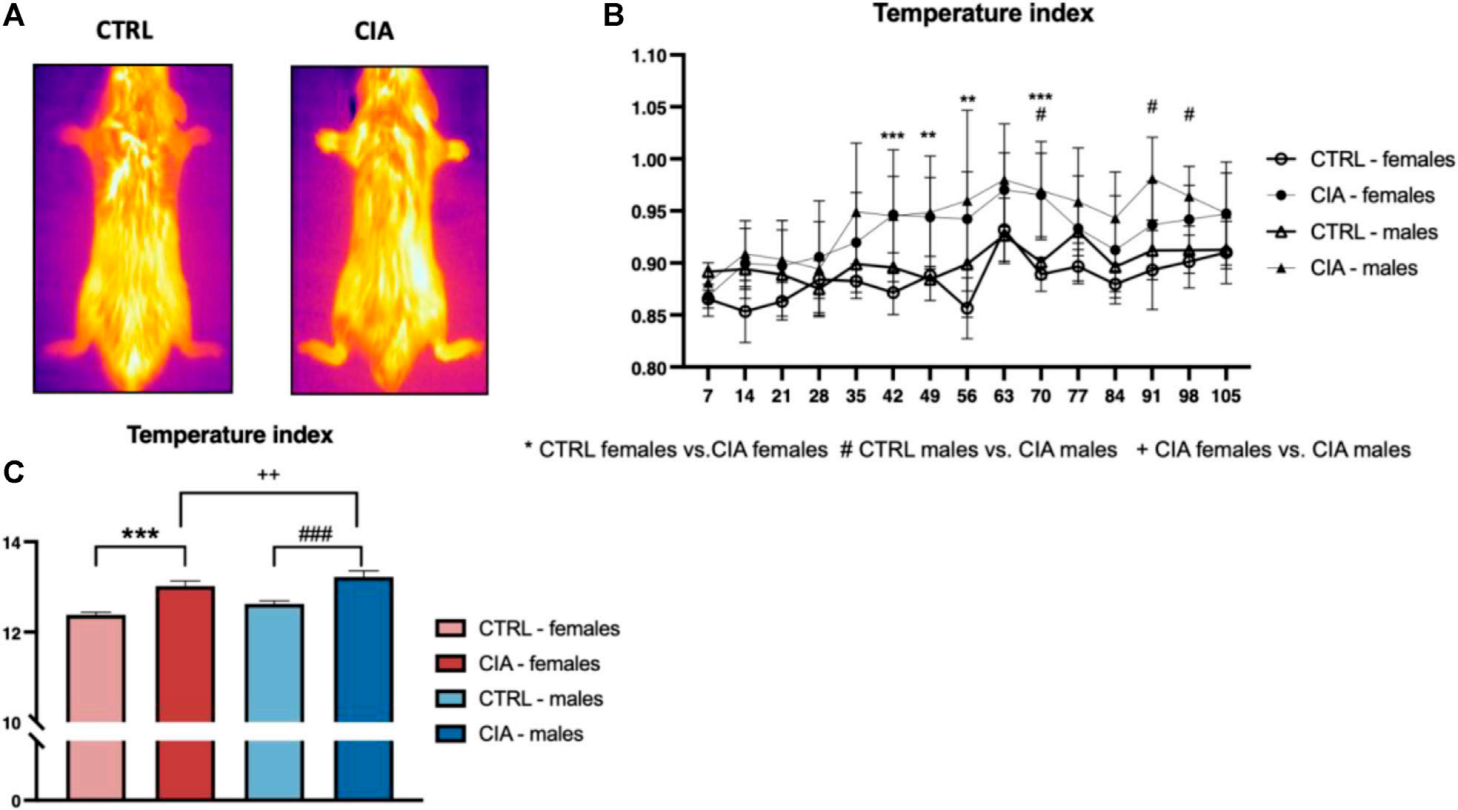
Dynamics of paw temperatures in the CIA model. **(A)**—Representative thermal images illustrating the temperature differences in the paws between a CTRL mouse and a CIA mouse with active arthritis. **(B)**—Dynamics of the temperature of paws regarding the sex and group of mice. **(C)**—Differences in the area under the curve of the temperature of paws during the experiment according to the sex and groups of mice. [CTRL-females (*n* = 5), CIA-females (*n* = 12), CTRL-males (*n* = 5), CIA-males (*n* = 9)). (*p* < 0.05 = *, *p* < 0.01 = **, *p* < 0.001 = ***, *p* < 0.05 = #, *p* < 0.01 = ##, *p* < 0.001 = ###, *p* < 0.05 = +, *p* < 0.01 = ++, *p* < 0.001 = +++).

Temperature index dynamics during the development of arthritis was affected by both group (F = 12.3; *p* < 0.001) and time (F = 6.19; *p* < 0.001) factors as well as their interaction (F = 1.62; *p* < 0.001). Higher temperatures were observed in CIA females compared to CTRL females on days 42, 49, 56, 70 after immunization with collagen (all: *p* < 0.01; Figure 2B). The CIA males displayed a higher temperature of paws compared to CTRL males on day 70, 91, and 98 after collagen injection (all: *p* < 0.05; Figure 2B). The One-way ANOVA from AUC of the dynamics of the temperature index showed significant differences between groups (F = 81.1; *p* < 0.001, Figure 2C). The CIA females as well as CIA males displayed higher temperature in the AUC compared to controls (females: CIA vs. CTRL: *p* < 0.001; males: CIA vs. CTRL: *p* < 0.001, Figure 2C). CIA males showed higher paw temperatures in the AUC of the temperature index compared to CIA females (*p* < 0.01; Figure 2C).

### 3.3 Structural changes in microCT bone density and functional consequences on locomotor activity

**FIGURE 3:**
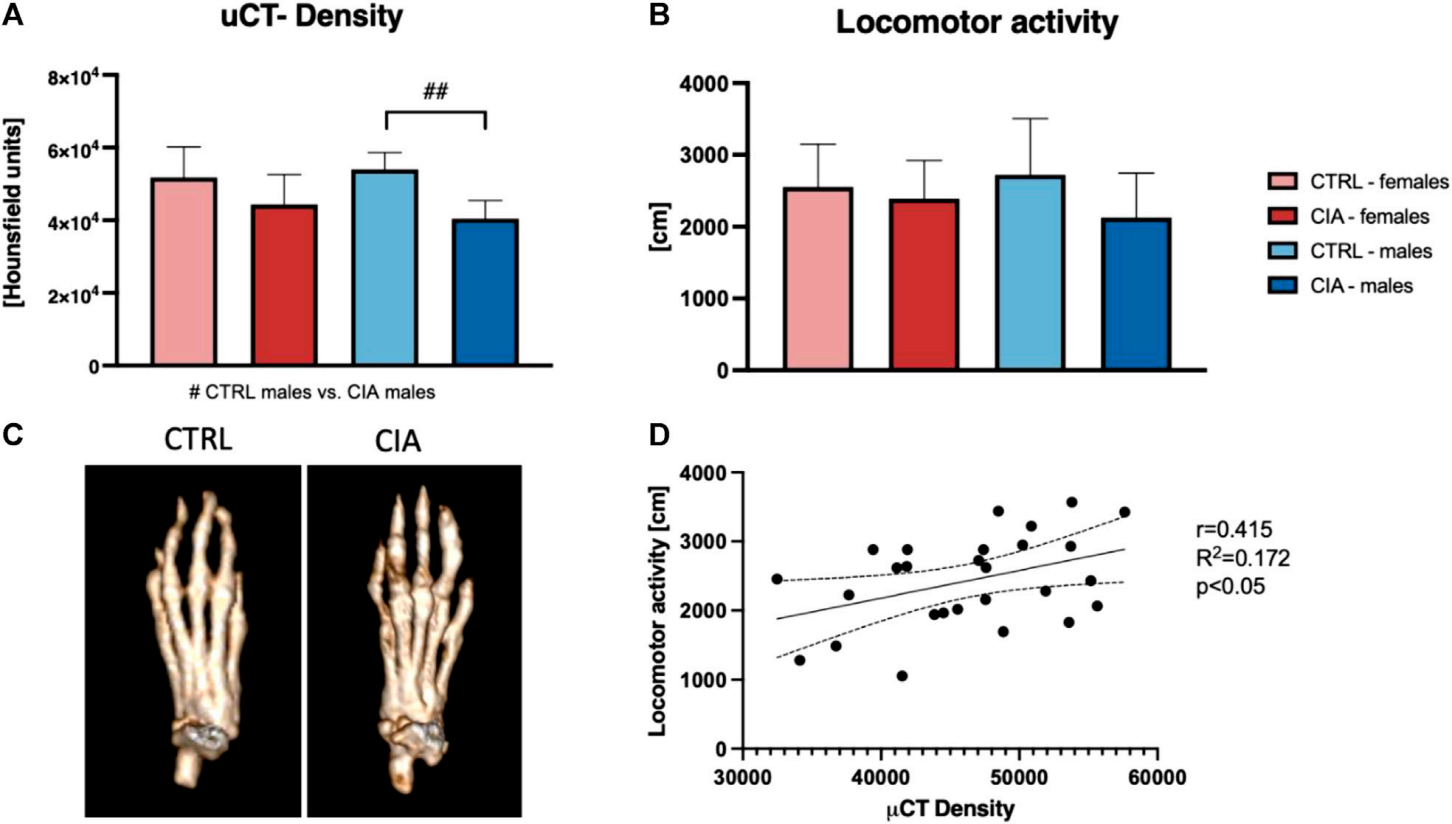
Long-term effects of the CIA model on the bones and locomotion. **(A)**—Analysis of periarticular bone density based on micro-CT. **(B)**—Results of the locomotor activity regarding the sex and the group of mice. **(C)**—Representative pictures showing the bone density as a long-term effect of the CIA model (*p* < 0.05 = #, *p* < 0.01 = ##, *p* < 0.001 = ###). **(D)**—Correlation between micro-CT density and locomotor activity (R2 = 0.172; *p* < 0.05). [CTRL-females (*n* = 5), CIA-females (*n* = 9), CTRL-males (*n* = 5), CIA-males (*n* = 9)].

To further investigate chronic damage to mouse joints and bones, Periarticular bone density was assessed at the end of the experiment using micro-CT. One-way ANOVA indicated significant differences between groups (F = 6.38; *p* < 0.01). The results indicated a 25.1% lower bone density in male CIA mice compared to their control group (*p* < 0.01). There were no significant differences between female CIA mice and their controls (p = ns) as well as no sex differences between CIA females and CIA males (p = ns; Figure 3A). To assess whether and to what extent CIA has an impact on the mouse joint in the long-term, an open-field test for locomotor activity was conducted at the end of the experiment. The results indicate no significant differences in locomotor activity between groups of mice (F = 1.163; p = ns; Figure 3B). After all, the correlation analysis between bone density and locomotor activity was conducted to elucidate functional consequences of arthritis. Results indicate a positive correlation between locomotor activity and bone density in CIA and control mice of both sexes (*R* ^2^ = 0.172; *p* < 0.05; Figure 3D).

### 3.4 Association between paw swelling and arthritis scores

**FIGURE 4:**
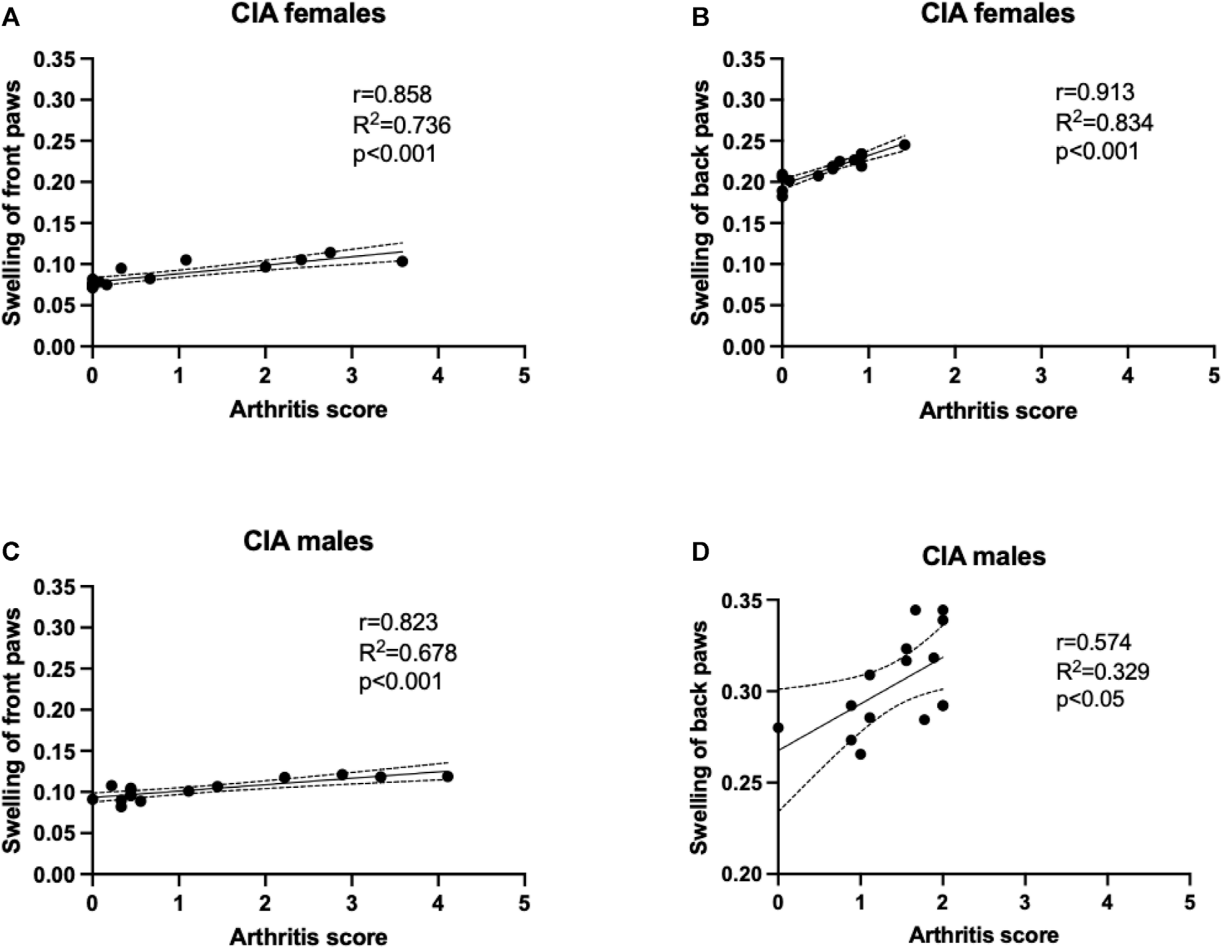
A correlation analysis was performed to show the relationship between arthritis scores and paw swelling, which was assessed using plethysmometry. **(A)**—Correlation of arthritis scores and swelling of front paws in CIA females mice. **(B)**—Correlation of arthritis scores and swelling of back paws in CIA females mice. **(C)**—Correlation of arthritis scores and swelling of front paws in CIA male mice. **(D)**—Correlation of arthritis scores and swelling of back paws in CIA male mice. [CIA-females (*n* = 12), CIA-males (*n* = 9)].

Besides the evaluation of arthritis scores, also weekly measurement of paw swelling using plethysmometer was conducted. The arthritis scores of front paws positively correlated with the swelling of the front paws in CIA females (*R* ^2^ = 0.736; *p* < 0.001; Figure 4A), as well as in CIA males (*R* ^2^ = 0.678; *p* < 0.05; Figure 4C). Back paws swelling positively correlated with the arthritis scores of back paws in CIA females (*R* ^2^ = 0.834; *p* < 0.001; Figure 4B), as well as in CIA males (*R* ^2^ = 0.329; *p* < 0.05; Figure 4D).

### 3.5 Urinary proteins and circulating sex hormones

**FIGURE 5:**
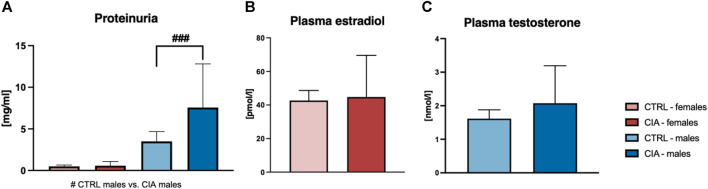
**(A)**—Quantification of proteinuria according to the sex and group of mice. [CTRL-females (*n* = 5), CIA-females (*n* = 12), CTRL-males (*n* = 4), CIA-males (*n* = 9)]. **(B)**—Quantification of plasma estradiol concentration in CTRL and CIA female mice. [CTRL-females (*n* = 5), CIA-females (*n* = 9)]. **(C)**—Quantification of plasma testosterone concentration in CTRL and CIA male mice. [CTRL-males (*n* = 4), CIA-males (*n* = 7)]. (*p* < 0.05 = #, *p* < 0.01 = ##, *p* < 0.001 = ###).

One-way ANOVA of results from measurements of urinary proteins showed significant differences (F = 6.38; *p* < 0.001). CIA males displayed higher concentrations of urinary proteins compared to CTRL males (*p* < 0.001; Figure 5A). No differences in urinary protein levels were seen between female CIA and CTRL female mice (p = ns; Figure 5A). Concentration of plasma estradiol did not differ between CIA females and CTRL females (t = 0.1819; p = ns; Figure 5B). Similarly, there were no significant differences in circulating testosterone concentrations of CIA males in comparison to CTRL males (*t* = 0.7911; p = ns; Figure 5C).

### 3.6 Histological assessment of joints

**FIGURE 6:**
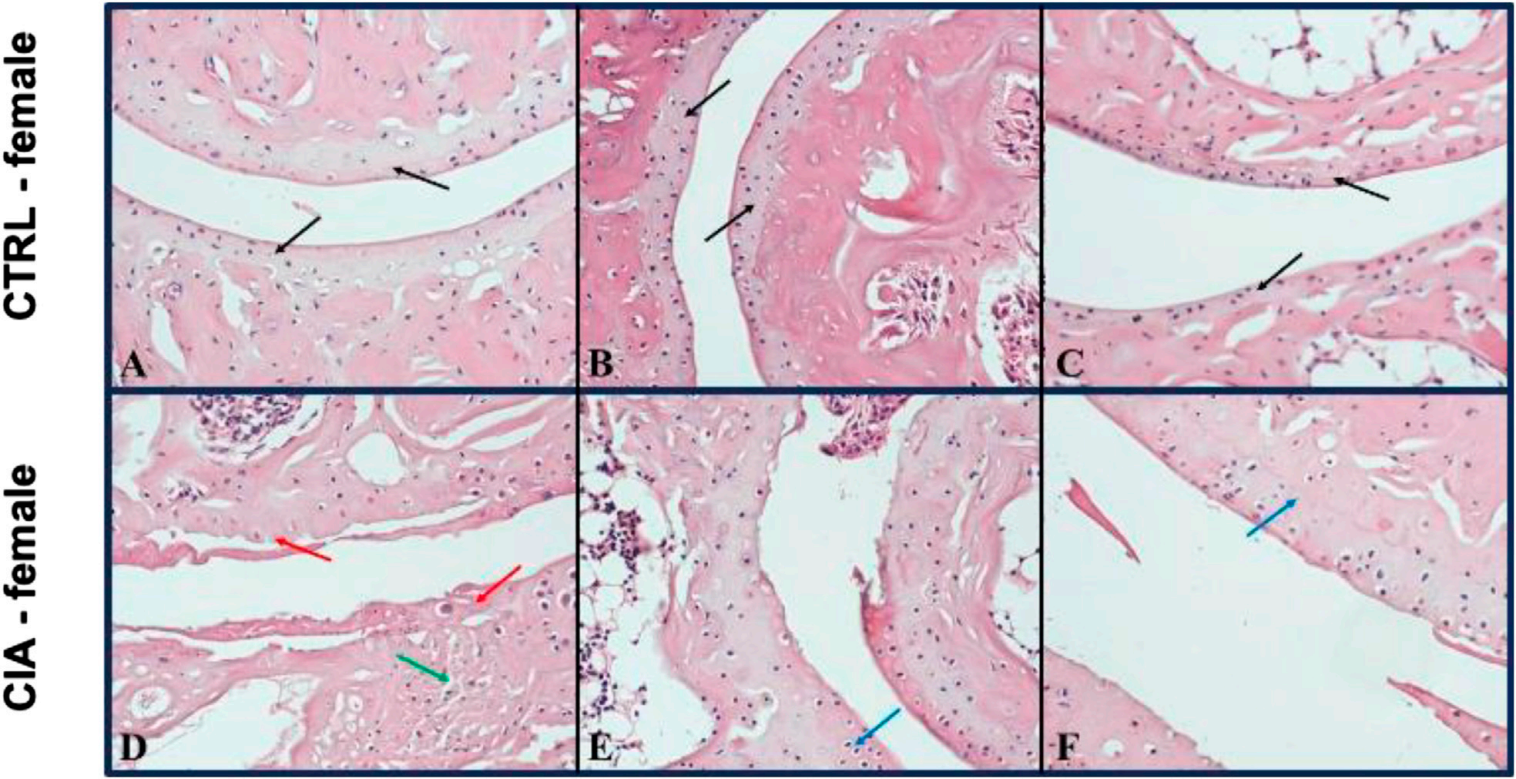
**(A–C)**—Representative pictures of histological evaluation of paw in a Ctrl female. **(D–F)**—Representative pictures of histological evaluation of paw in a CIA female. A histologically normal layer of hyaline cartilage in the superficial bone joints in control females [**(A–C)** black arrows], while in CIA females, an atrophic residual layer of hyaline cartilage with some residual disintegrating pinkish tissue [**(D)** red arrow], including a preserved layer of hyaline cartilage with compensatory hyperplasia of thinned, variably sized groups of chondrocytes was observed [**(E, F)** blue arrows]. HE: 200x.

**FIGURE 7:**
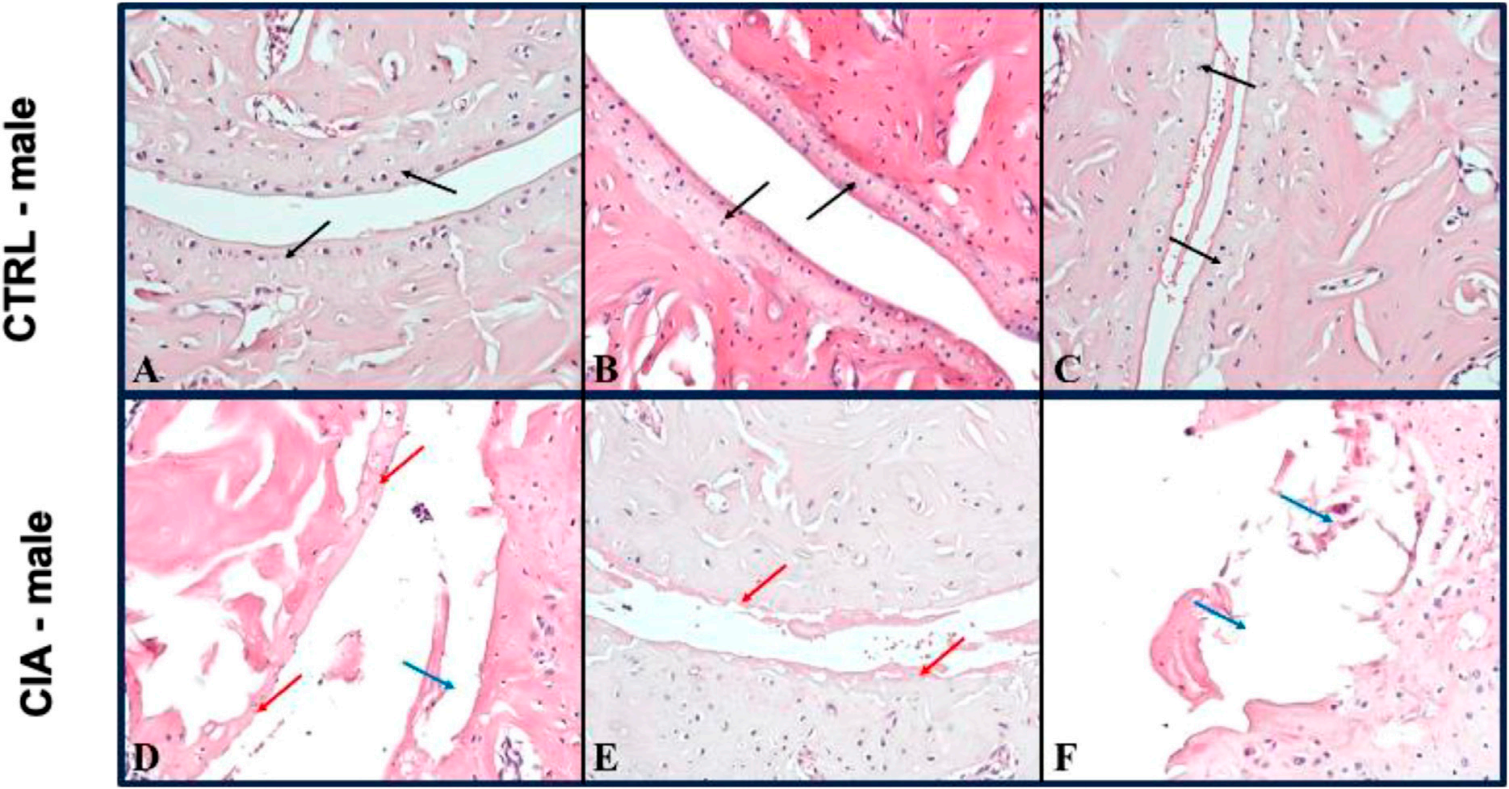
**(A–C)**—Representative pictures of histological evaluation of paw in a CTRL male. **(D–F)**—Representative pictures of histological evaluation of paw in a CIA female. In control males, in the joints of long and short bone, a 5/5 continuous, equally thick layer of normal hyaline cartilage was observed [**(A–C)** black arrows]. In CIA males, an atrophic residual discontinuous layer of hyaline cartilage with individual chondrocytes [**(D)** red arrow], cartilage erosions [**(D)** blue arrow], or only some residual thin cartilage with disintegrating pinkish material [**(E)** red arrow], and focally cartilage erosions with disintegrated tissue was detected [**(F)** blue arrow]. IIE: 200x.

**FIGURE 8:**
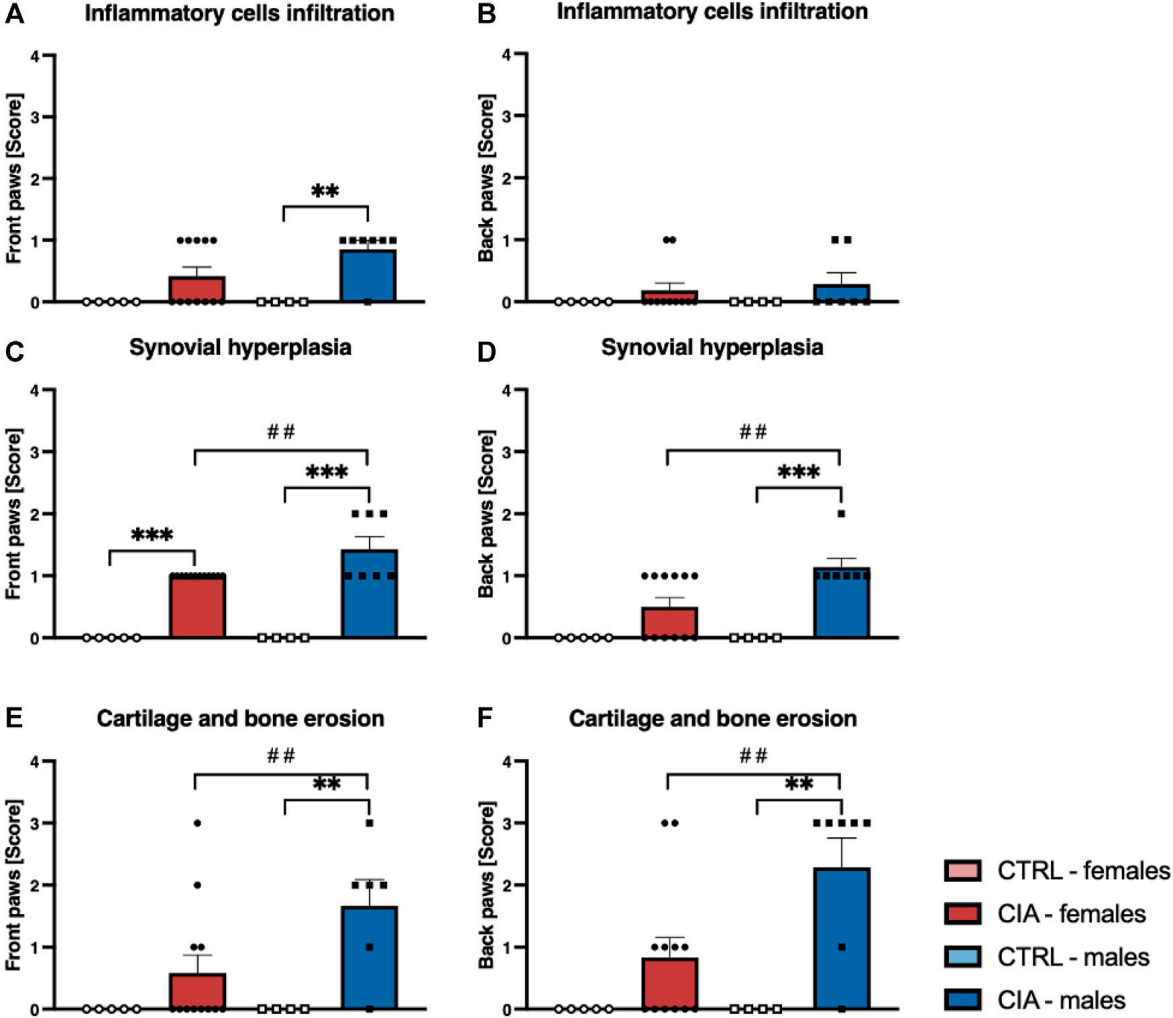
**(A)**—Inflammatory cells infiltration in joints of front paws according to the sex and group of mice. **(B)**—Inflammatory cells infiltration in joints of back paws according to the sex and group of mice. **(C)**—Synovial hyperplasia in joints of front paws according to the sex and group of mice. **(D)**—Synovial hyperplasia in joints of back paws according to the sex and group of mice. **(E)**—Cartilage and bone erosion in joints of front paws according to the sex and group of mice. **(F)**—Cartilage and bone erosion in joints of back paws according to the sex and group of mice. [CTRL-females (*n* = 5), CIA-females (*n* = 12), CTRL-males (*n* = 4), CIA-males (*n* = 7)]. (*p* < 0.01 = **, *p* < 0.001 = ***, *p* < 0.01 = ##).

Arthrosis was observed in 90% of male CIA mice (6/7), while in 50% (6/12) of female CIA mice. CIA females (histological picture Figures 6D–F) and CIA males (histological picture Figures 7D–F) showed a histologically well visible atrophy of hyaline cartilage with digested or disintegrated cartilaginous tissue on epiphysis and short bone surfaces in comparison to control mice (histological picture Figures 6A–C, histological picture Figures 7A–C). In CIA mice, besides focal compensatory hyperplasia of chondrocytes, the presence of irregularly shaped groups of chondrocytes and groups of apparently digested chondrocytes, including the presence of groups of semivital to necrotic chondrocytes were observed (histological picture Figures 6D–F, histological picture Figures 7D–F). In both, CIA females and CIA males, higher inflammatory cell infiltration of synovial membrane leading to mild synovial membrane thickening was shown (Figures 8A,B). However, it reached statistical significance only on the front paws (sex: F = 1.82; p = ns; treatment: F = 15.3; *p* < 0.01) of CIA males compared to control males (t = 3.45; *p* < 0.01; Figure 8A). There were no significant differences in inflammatory cell infiltration in back paws between CIA and CTRL females or males (sex: F = 0.12; p = ns; treatment: F = 2.4; p = ns; Figure 8B). Synovial hyperplasia in front and back paws was not affected by sex (front paws: F = 3.8; p = ns; back paws: F = 3.8; p = ns), but a main effect of CIA treatment on synovial hyperplasia of both paws was observed (front paws: F = 122; *p* < 0.001; back paws: F = 24.8; *p* < 0.001). CIA males showed a significantly higher degree of synovial hyperplasia compared to control male mice in both, front (t = 8.53; *p* < 0.001; Figure 8C) and back paws (t = 4.55; *p* < 0.001; Figure 8D), while in CIA females higher degree of the synovial hyperplasia was observed only on the front paws in comparison to control females (t = 7.03; *p* < 0.001; Figure 8C). CIA males displayed higher degrees of synovial hyperplasia compared to CIA females in both front (t = 3.37; *p* < 0.01; Figure 8C), and back paws (t = 3.72; *p* < 0.001; Figure 8D). Cartilage and bone erosion in both front and back paws were not affected by sex (front paws: F = 3.24; p = ns; back paws: F = 3.25; p = ns), but were significantly affected by CIA in both paws (front paws: F = 11.89; *p* < 0.001; back paws: F = 15; *p* < 0.001). Significantly higher cartilage and bone erosion was found only in CIA males, on both, front (t = 3.44; *p* < 0.01; Figure 8E) and back paws (t = 3.72; *p* < 0.01 Figure 8F) compared to control males. In females, CIA did not lead to significant cartilage and bone erosion either in front (*t* = 1.27; p = ns; Figure 8E) or back paws (*t* = 1.6; p = ns; Figure 8F). CIA males showed higher degrees of bone and cartilage erosion compared to CIA females in both, front (t = 3.11; *p* < 0.01; Figure 8E), and back paws (*t* = 3.12; *p* < 0.01; Figure 8F).

### 3.7 Plasmatic inflammatory cytokines

Plasma TNFα concentrations did not differ between CIA and CTRL males and females (sex: F = 1.99; p = ns; treatment: F = 0.11; p = ns; Supplementary Figure S1A). Regarding plasma IL-1α concentrations, two-way ANOVA revealed a main effect of sex (F = 4.25; *p* < 0.05), but not CIA treatment (F = 4.25; p = ns). Following the Bonferroni *post hoc* correction test, there were no significant differences between CTRL (t = 2.19; *p* = 0.07) and CIA (t = 0.46; p = ns) females and males (Supplementary Figure S1B). Circulating IL-10 concentrations were not affected by either sex (F = 2.37; p = ns) or CIA treatment (F = 0.44; p = ns, Supplementary Figure S1C). Plasma IL-6 concentrations were affected by CIA treatment (F = 5.5; *p* < 0.05), but not by sex (F = 0.37; p = ns). Bonferroni *post hoc* correction did not show significant differences between CTRL (females vs. males: *t* = 0.39; p = ns) and CIA (females vs. males: t = 0.51; p = ns) females and males (Supplementary Figure S1D). Differences in circulating concentrations of IL-1β were not observed (sex: F = 2.25; p = ns; treatment: F = 0.17; p = ns; Supplementary Figure S1E). Plasma concentrations of IL-23 did not differ between CIA and CTRL females and males (sex: F = 3.11; p = ns; treatment: F = 0.052; p = ns; Supplementary Figure S1F). Concentrations of circulating INF-ℽ was not affected by CIA treatment (F = 2.38; p = ns), or by sex (F = 0.172; p = ns; Supplementary Figure S1G). Plasma concentrations of INF-β did not differ between CIA and CTRL females and males (sex: F = 0.81; p = ns; treatment: F = 0.41; p = ns; Supplementary Figure S1H). Plasma concentrations of GM-CSF were similar between females and males after CIA treatment and in control animals (sex: F = 2.47; p = ns; treatment: F = 0.274; p = ns; Supplementary Figure S1I). Concentrations of circulating IL-17A, IL-27, and MCP-1 did not differ between CIA and CTRL females and males (IL-17A: sex: F = 2.02; p = ns; treatment: F = 0.121; p = ns; Supplementary Figure S1J; IL-27: sex: F = 2.00; p = ns; treatment: F = 0.374; p = ns; Supplementary Figure S1K; MCP-1: sex: F = 3.02; p = ns; treatment: F = 0.133; p = ns; Supplementary Figure S1L).

## 4 Discussion

### 4.1 Dynamics of arthritis in the CIA model

In this study, long-term effects of CIA on the joints of middle-aged mice of both sexes were observed. To ensure a reliable comparison between groups and reduce the effect of age-related changes on the organism and joints, aged matched control mice of both sexes were used and followed longitudinally. Weekly measurements revealed a comparable incidence but earlier onset of arthritis in male mice. In addition, a higher incidence and severity of arthritis in the front paws compared to the back paws of mice of both sexes were found. Male mice achieved the maximum arthritis score 1 week earlier than females. At the end of the experiment, four male and one female mouse still showed low but evident signs of arthritis. To date, only one other study has examined the long-term dynamics of the CIA model in both sexes of mice (Holmdahl et al., 1985). The study (Holmdahl et al., 1985), found that only 6% of female mice but 91% of male mice developed signs of arthritis during a 20-week course post-immunization with bovine collagen II. Furthermore, Holmdahl et al. also demonstrated that female sex hormones suppress the development of CIA, by showing that ovariectomized female CIA mice display a significantly higher incidence and severity of arthritis compared to non-ovariectomized female CIA mice, whereas orchidectomized male mice developed arthritis with an incidence and severity that did not differ significantly from non-castrated male CIA mice (Holmdahl et al., 1986). We observed an earlier onset of arthritis in male mice compared to female mice, and a higher incidence and severity of arthritis in the front paws of both sexes with CIA. However, it is important to note that the kinetics of arthritis development may differ from one experiment to another, and there is a lack of comparable data on the onset of arthritis between sexes in the CIA model (Brand et al., 2007). The differences in the dynamics, incidence, and severity of arthritis between male and female mice in our study and those of Holmdahl et al., in which young mice (7–10 weeks old) were used, may be related to physiological changes associated with aging, particularly changes in sex hormone concentrations, as research suggests that mice undergo the equivalent of human perimenopause by the age of 9 months (Brinton, 2012). However, the matter of sexual dimorphism in the CIA model remains a subject of debate, with conflicting findings suggesting predominantly a lower occurrence of arthritis in female mice (Holmdahl et al., 1986; 1992; Kim and Kim, 2020; Luan et al., 2021) but some proposing no disparities between sexes (Brand et al., 2007; De Lima Xavier et al., 2012). It should also be noted, that the evaluation of arthritis dynamics, typically performed through visual scoring on a weekly basis, is subjective and can be influenced by observer bias, highlighting the necessity for supplementary measurements such as Plethysmometer and IRT, which have not been adopted, especially in early studies investigating sex differences in the CIA model (Holmdahl et al., 1985; 1986). Furthermore, it has to be considered that male DBA/1J mice over the age of 4 months are stated to be susceptible to the development of spontaneous arthritis, which is believed to be caused by external stress and sensory factors (Braem et al., 2012). Although none of the control mice in our study displayed any signs of arthritis, we cannot rule out the possibility that it may have contributed to the incidence of arthritis in male CIA mice, since the two types of arthritis are macroscopically indistinguishable from each other (Braem et al., 2012). To conclude, the influence of various factors, such as observer bias, age, sex, genetic variations in DBA/1J mice and their sub-strains, type and source of collagen, stress, environmental and housing conditions, and diet, may impact and distort the course and incidence of experimental arthritis (Asquith et al., 2009; Braem et al., 2012; Bai et al., 2021; Wang et al., 2021). Although, many of these variables, such as genetics, age, sex, diet, and environmental factors (e.g. stress) are risk factors in the pathogenesis of RA as well, they need to considered when conducting experiments in the CIA model (Brand et al., 2007; Asquith et al., 2009; Smolen et al., 2018; Romão and Fonseca, 2021).

### 4.2 IRT as a novel imaging method in RA and the CIA mouse model

In inflammatory processes, vessel dilatation causes increased blood flow and, thereby, an elevation of temperature of the inflamed tissue (Claesson-Welsh, 2015). Therefore, thermal imaging, displaying the heat radiation of objects, offers a potential non-invasive and cheap method to evaluate the inflammation of tissues (Pauk et al., 2019; Schiavon et al., 2021). While it is already under investigation as an additional imaging method in RA patients (Kow and Tan, 2023), to date, only one published study (Nosrati et al., 2020) has used thermal imaging in the CIA mouse model as a novel modality, to monitor disease activity in the paws of mice. Similarly, we used a thermal camera to evaluate the temperature of the paws, and a thermal index to normalize the temperatures of paws to the average reference temperature (Nosrati et al., 2020). In our experiment, the elevation of paw temperature was consistent with increasing and achieving maximal arthritis scores in both sexes of mice. Interestingly, the temperatures of the paws of male CIA mice remained at a higher level throughout the decline in the arthritis scores. While there is no comparable data for this long-term rise in paw temperature, it could be hypothesized that while the arthritis activity decreases, the vessel dilation might still be present. On the other hand, as angiogenesis plays an important role in the pathogenesis of RA and anti-angiogenic therapy has been proven to be a promising target in the CIA model, a potentially higher density of vasculature, caused by prior inflammation, might lead to this persistent higher paw temperature (Paleolog, 2002; Elshabrawy et al., 2015).

### 4.3 Long-term effects of CIA

To investigate the long-term effects of CIA in middle-aged mice of both sexes several measurements were conducted at the end of the experiment. This included an open-field test, micro-CT, joint histology, measurement of proteinuria as well as assessment of plasma concentrations of sex hormones and pro-inflammatory cytokines of mice. Although no significant differences were seen in systemic pro-inflammatory cytokines between CIA mice and their control groups, which aligns with the low arthritis scores the mice received at the end of the experiment, notable, long-term consequences were evident, especially in the male CIA mice including damage to joints, lower bone density and potential kidney damage.

#### 4.3.1 Histology of joints

Inflammatory cell infiltration, synovial hyperplasia as well as cartilage and bone erosions are characteristics of joint damage shared by both RA patients and mice in the CIA model (Williams, 2004; Schett & Gravallese, 2012). To assess these characteristics, histology of joints in CIA mice is usually conducted at the peak of paw inflammation and arthritis incidence, which occurs around the sixth to eighth week following CIA induction (Brand et al., 2007; Luan et al., 2021). However, to assess the long-term effects of CIA on the mouse joints, in this experiment, histopathological examination of front and back paws was conducted after day 105. Arthrosis was observed in 90% of male CIA mice (6/7), while in female CIA mice (6/12) the incidence of arthrosis was 50%. The arthrosis was characterized by visible atrophy of the hyaline cartilage accompanied by the presence of digested or disintegrated cartilaginous tissue at various locations on the surface of epiphysis and junctions of short bones. Additionally, in both CIA males and females, focal compensatory hyperplasia of chondrocytes was observed, along with irregularly shaped groups of chondrocytes and groups of chondrocytes that exhibited signs of diminished vitality or necrosis. Furthermore, a mild thickening of the synovial membrane was detected in both CIA females and males. A scoring system was employed to evaluate the extent of joint damage, encompassing parameters such as inflammatory cell infiltration, synovial hyperplasia, as well as cartilage and bone erosion (Li et al., 2021). The male CIA mice exhibited a significantly higher degree of synovial hyperplasia as well as cartilage and bone erosions compared to the female CIA mice. Inflammatory cell infiltration was higher in CIA males compared to control males, but this effect was observed only on the front paws. These findings suggest a significant long-term impact of CIA on the joints of mice of both sexes, however, more profound damage of joints was seen in the male CIA mice compared to the female CIA mice.

#### 4.3.2 Periarticular bone density and locomotor activity

OP is a frequent complication seen in patients with RA, especially in women in post-menopausal age (Llorente et al., 2020; Moshayedi et al., 2022). Although the pathogenesis of RA-related OP is uncertain, it is evident that the RANK/RANKL/OPG and Wnt/DKK-1/sclerostin pathways and certain proinflammatory cytokines are crucial in its development (Llorente et al., 2020). Micro-CT provides a non-invasive approach to evaluate alterations in bone structure in the CIA mouse model (Yang et al., 2013). While the method is most often deployed at the peak of inflammation, which is around the sixth-eighth week post-immunization, the present study offers data from mice in which obvious inflammation has already decreased. Trabecular bone density inside the second metatarsal head of the back paws was significantly lower in male CIA mice, indicating a more severe course of arthritis in the long-term in males. Moreover, deformities similar to those observed in patients with RA have been observed in both sexes of mice (Jacobson et al., 2008).

In the present study, no significant differences in the locomotor activity of mice were found. In contrast, other studies that assessed the locomotor activity of mice in the CIA model within the first 3 weeks after the booster immunization showed a worsening in mice mobility, which is stated to correlate with arthritic scores (Hartog et al., 2009; Marinov et al., 2022). However, in the present study, when comparing the individual locomotor activity of mice to their trabecular bone density, a positive correlation was observed, indicating worsening of mobility in mice with lower bone density. Therefore, our study suggests that long-term effects of CIA on the joints of mice, in terms of locomotion, is connected to the individual course of the disease of each mouse, rather than to their sex.

#### 4.3.3 Proteinuria in male CIA mice

Proteinuria can be used as a sign of kidney disease in patients with RA (Niederstadt et al., 1999). In addition, a previous study has found higher urine protein concentrations and lower renal function in CIA rats (Wang et al., 2020). Therefore, in the present study, urinary protein concentrations were also assessed and a significantly higher proteinuria in male CIA mice compared to CTRL mice was observed, suggesting kidney damage. However, in general, female mice excrete less protein than male mice and DBA/1 mice are susceptible to immune-mediated nephritis leading to marked proteinuria (Xie et al., 2004; Wicks, 2016). This susceptibility is thought to be linked to their expression of interleukin-1 (IL-1), a pro-inflammatory cytokine that also plays a pivotal role in arthritis development in both, patients with RA and mice with CIA (Dayer, 2003; Xie et al., 2004; Kim & Kim, 2020). Based on our findings, it is possible that CIA could play a role in the initiation but also the progression of kidney disease in DBA/1J mice. Nevertheless, additional research is needed to understand the underlying mechanisms.

#### 4.3.4 The interplay between sex hormones and disease in RA and the CIA mouse model

The modulatory effects of sex hormones on the immune system are pleiotropic and have been described to affect both, the adaptive and the innate immune defense (Taneja, 2018). Therefore, the sex-based disparity in autoimmune disease is thought to be linked not only to the X chromosome, but also to the immunomodulatory effects of sex hormones (Moulton, 2018). RA predominantly affects women at an estimated female:male ratio of 3:1, with a peak incidence coinciding with the onset of menopause, when circulating estrogen concentrations decrease, suggesting a protective role of estrogens (Taneja, 2018). Moreover, the symptom improvement observed in pregnant women with RA is believed to be linked to elevated sex hormone concentrations during pregnancy (Hazes et al., 2011). The effects of sex hormones on the immune system have also been reported to affect the severity and incidence of experimental arthritis, as ovariectomized female mice displayed significantly higher severity and incidence of CIA than non-ovariectomized controls (Holmdahl et al., 1986). Although the exact mechanism is unknown and most likely multifactorial, it has been shown that supplementation with ethinyl estradiol suppresses clinical and histological CIA symptoms by inhibiting the production of tumor necrosis factor α and IL-1β (Subramanian et al., 2005). In men, higher susceptibility to RA has been proposed to be associated with a decline in bioavailable testosterone concentrations, although it is uncertain whether this is a result or a contributing factor to the disease, given that low testosterone concentrations have been found to correlate with disease activity (Tengstrand et al., 2002). However, orchiectomized male mice in the CIA model develop arthritis with an incidence and severity that does not differ significantly from non-castrated male CIA mice (Holmdahl et al., 1986). To determine whether CIA had a lasting impact on circulating sex hormones, plasma testosterone in male mice and estrogen concentrations in female mice were measured at the end of the experiment. No differences between CIA mice and control mice were observed in either of the sexes. However, these results do not exclude the possible effects of lower sex hormone concentrations in aged female mice on the development or severity of experimental arthritis, as female mice undergo perimenopause at around 9 months of age (Brinton, 2012). Although a reduction in sex hormones with age in DBA/1J mice is likely, a limitation of this study is the lack of comparable data on the dynamics of circulating sex hormone concentrations throughout the lifespan in this type of mouse strain.

### 4.4 Sex differences in RA and the CIA mouse model

RA exhibits a peak incidence around the age of 50 years, with women being 2–3 times more likely to develop the condition compared to men (Gerosa et al., 2008; van der Woude & van der Helm-van Mil, 2018). In the CIA mouse model on the other hand, sex differences have been predominantly described as male mice exhibiting a higher incidence and severity of arthritis symptoms compared to female mice (Holmdahl et al., 1986; Kim & Kim, 2020; Luan et al., 2021; Marinov et al., 2022). However, there is a notable gap of data pertaining to the application of this model in mice at the age that mimics the typical onset age of human RA and data regarding the long-term effects of the CIA model is also lacking in scientific literature. Therefore, our study aimed to replicate the peak onset age observed in human RA by using middle-aged mice of both sexes in the CIA mouse model, to see whether this will lead to a shift in incidence and severity of arthritis symptoms and long-term effects on mice joints to the female side. Surprisingly, while the incidence of arthritis symptoms was similar in both sexes, an earlier onset of arthritis and more severe long-term effects of CIA on joints were observed in middle-aged male CIA mice, which do not recapitulate the situation observed in human RA. The underlying mechanisms responsible for the observed sex differences in the CIA model in mice and humans with RA remain elusive (Kovacs and Olsen, 2011; Smolen et al., 2018; Marinov et al., 2022). However, despite the contrasting sex differences between human RA and the CIA mouse model, it is believed that in both, variations within the sex chromosomes and the influence of sex hormones on the immune system play a pivotal role in shaping the observed disparities between the sexes (Holmdahl et al., 1986; Gerosa et al., 2008; Taneja, 2018). Additionally, several risk factors that have been described for human RA and CIA mice, were already observed to vary between the sexes of mice, such as the composition of the gut microbiota, individual responses to stress and levels of physical activity, and therefore might have the potential to influence both the occurrence and severity of experimental arthritis between sexes (Braem et al., 2012; Rosenfeld, 2017; Bai et al., 2021; Romão & Fonseca, 2021; Takahashi, 2021; Shobeiri et al., 2022). Therefore, our study not only sheds light on a limitation of the CIA model in mice when compared to human RA, but also serves as a fundament for using animals of both sexes at clinical relevant age in the CIA model. Further experimental studies are needed to unravel the complex interactions between age, sex and immune response in mice which might be relevant for striving towards more targeted and effective interventions for both male and female patients with RA.

### 4.5 Limitations and similarities of the CIA mouse model to RA

Our hypothesis, that the female sex will worsen the progress of the CIA model and increase the risk of long-term consequences in middle-aged mice, was not confirmed. Furthermore, compared to the modeled disease and likewise observed in our experiment, CIA lacks real chronicity. However, while there are inconsistencies regarding arthritis incidence in female mice, in the present study a satisfactory incidence of CIA in both male and female middle-aged mice was observed, which could prove valuable for future experiments, particularly those exploring the relationship between age and sex to the actual disease (Holmdahl et al., 1985; Brand et al., 2007; Luan et al., 2021; Marinov et al., 2022). To determine whether a shift in arthritis incidence among DBA/1J mice in the CIA model with an increase in age is possible, more consistent data is needed not only in young but especially in aged mice. Moreover, to enhance the comparability of the CIA model, it is important to consider but also describe different factors that may influence the occurrence and severity of arthritis and to also deploy, besides arthritis scoring, various additional assessment methods for the evaluation of arthritis. However, similar to humans diagnosed with RA, mice in the CIA model exhibit features such as proliferative synovitis with infiltration of activated T Cells and granulocytes, cartilage degradation, pannus formation, and bone erosion (Williams, 2004). In addition, certain expressions of MHC class 2 allotypes correlate with susceptibility to the disease, and inflammatory cytokines can be found in higher concentrations in both, joints of mice in the CIA model and RA patients (Holmdahl et al., 1989; Brennan & McInnes, 2008). In summary, CIA is a robust and replicable animal model of RA but limited by certain factors that must be considered before its usage. Therefore, our research aims to provide further insights and limitations of this model and to encourage the adoption of novel techniques such as IRT, as well as the investigation into the impact of sex, age, and comorbidities in animal models of RA.

### 4.6 Limitations of this study

Besides its strengths, the present study also faces several limitations. First, only the CIA model in mice was investigated, which may not be representative of other animal arthritis models. Additionally, comparing this study with others is challenging due to the limited availability of long-term data on the effects of CIA in mice, as well as the absence of data in aged mice and inconsistencies in young mice. Therefore, caution should be taken when generalizing our findings and young male mice should potentially be preferred for testing novel therapeutic strategies in the CIA mouse model. Moreover, the study did not evaluate the biochemical markers of local or systemic inflammation at various time points of the experiment, which could have provided more insight into the underlying mechanisms of arthritis in the CIA model, and therefore could limit our understanding of the complex interplay between the aging organism, sex hormones, inflammation, and the development of CIA. Therefore, further research incorporating multiple models as well as biochemical evaluation of inflammation at various time points is necessary to better understand the role of sex and age differences in the development, progression and long-term consequences of experimental arthritis models.

### 4.7 Conclusion

In conclusion, to the best of our knowledge, this is the first study to analyze sex differences in the CIA mouse model in middle-aged mice over the long-term. Our results indicate a similar incidence of arthritis in both sexes of mice, with an earlier onset in males. In the case of long-term consequences, male mice with CIA exhibited more severe joint damage, lower bone density and higher proteinuria compared to their controls and female mice with CIA. However, the underlying immune mechanisms not analyzed in this study require further investigation.

## Data Availability

The raw data supporting the conclusion of this article will be made available by the authors, without undue reservation.
