# Supplementary material for: Sex differences in long-term effects of collagen-induced arthritis in middle-aged mice
Source: Front Physiol. 2023 Jun 28;14:1195604. doi: 10.3389/fphys.2023.1195604 (PMC10337783; doi:10.3389/fphys.2023.1195604)
Supplement: Supplementary file 1 [file DataSheet1.docx]

Supplementary Material

**Sex differences in long-term effects of collagen-induced arthritis in middle-aged mice**

**Bernhard Maximilian Schuh^1^, Kristína Macáková^1^, Andrej Feješ^1^, Tim Groß^1^, Paulína Belvončíková^1^, Jakub Janko^1^, Dominik Juskanič^2,3^, Samuel Hollý^4^, Veronika Borbélyová^1^, Emőke Šteňová^5^, Michal Pastorek^1^, Barbora Vlková^1^, Peter Celec^1,6*^**

*** Correspondence:**assoc. prof. Peter Celec MD, Dipl Ing., Dr. Rer. Nat., DSc., MPH

# Supplementary Tables

| **Pathology** | **Classification score** | **Histological view** |
| --- | --- | --- |
| Inflammatory cells infiltrate | 0 | no infiltrate detected |
|  | 1 | modest leucocyte infiltration in synovial tissue, no fluid leucocytes |
|  | 2 | moderate leucocyte infiltration in synovial tissue and in fluid phase, with loss of synovial architecture |
|  | 3 | gross leukocytes infiltration in synovial membrane and fluid space, significant loss of synovial articular architecture |
| Synovial hyperplasia | 0 | no abnormalities detected |
|  | 1 | synovial lining layer 2-4 cells thick |
|  | 2 | synovial lining layer 5 and more cells thick associated with moderate expansion of the sub-lining layer |
|  | 3 | synovial lining layer 5 and more cells thick associated with significant expansion of the sub-lining layer zone and potentially with loss of synovial architecture |
| Erosion of cartilage and bone | 0 | no abnormalities |
|  | 1 | fibrillation of cartilage and/or mild erosive infiltration of periosteal and subchondral bone, nuclei intact within lacunae |
|  | 2 | moderate fibrillation and loss of cartilage and/or moderate erosive infiltration of periosteal and subchondral bone |
|  | 3 | significant loss of cartilage and/or erosive infiltration of periosteal and subchondral bone, nuclei show apoptosis within lacunae across a wide area of cartilage/bone |

## Table Supplementary 1. Methodology of histological scoring system

# Supplementary Figures


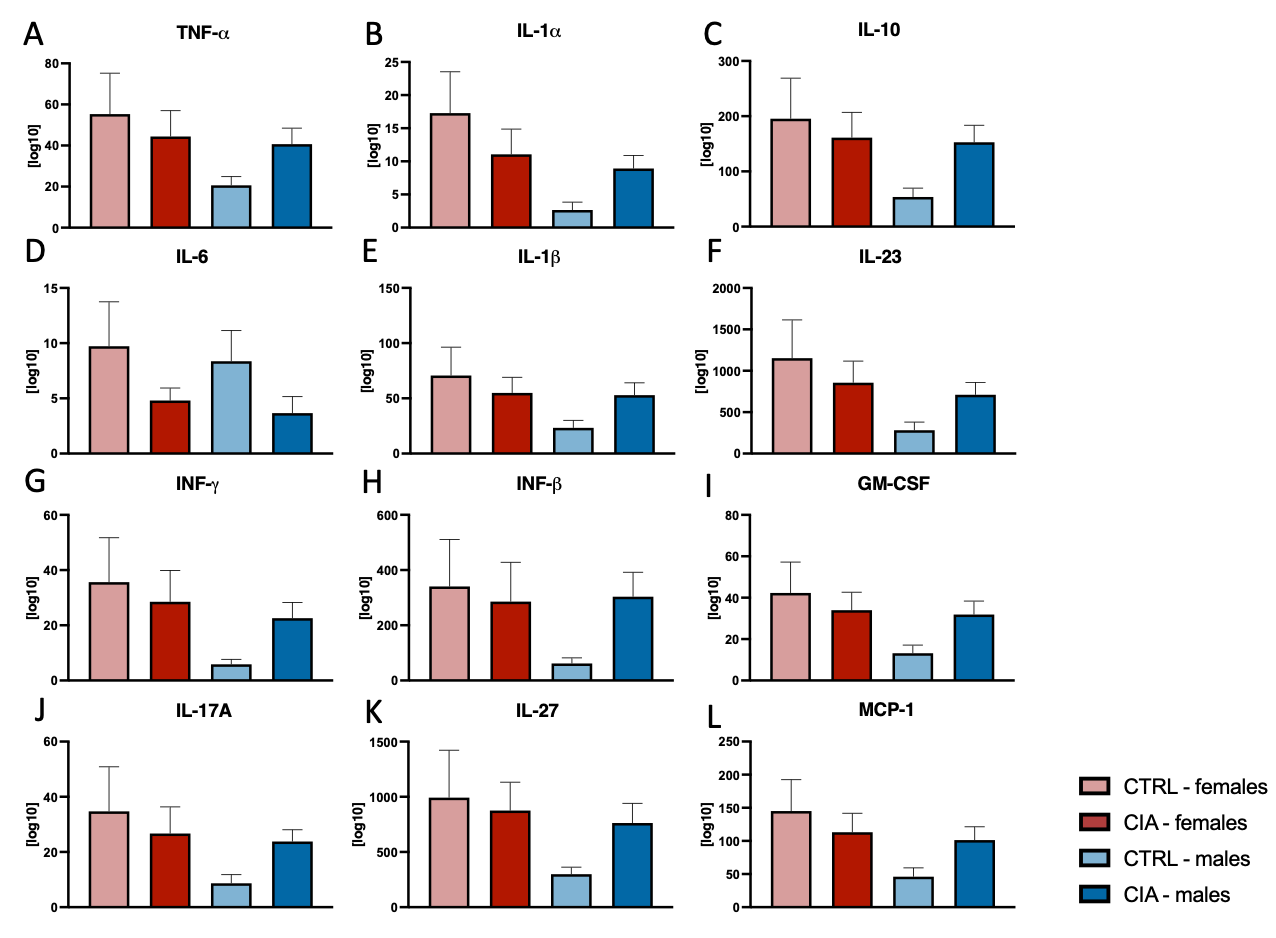


**FIGURE Supplementary 1.** Plasma concentrations of **(A)** tumor necrosis factor alpha (TNF-a), **(B)**
interleukin 1 alpha (IL1-a), **(C)** interleukin 10 (IL-10), **(D)** interleukin 6 (IL-6), **(E)** interleukin 1 beta (IL-1β), **(F)** interleukin 23 (IL-23), **(G)** interferon gamma (INF-𝛾), **(H)** Interferon beta (INF-β), **(I)** granulocyte-macrophage colony-stimulating factor (GM-CSF), **(J)** interleukin 17A (IL-17A), **(K)** interleukin 27 (IL-27), **(L)** monocyte chemoattractant protein-1 (MCP-1), in the experimental groups of mice at the end of the experiment. (CTRL-females (n=5), CIA-females (n=12), CTRL-males (n=5), CIA-males (n=9)). (p < 0.01 = **, p < 0.001 = ***, p < 0.01 = ##).
